# Gain of PITRM1 peptidase in cortical neurons affords protection of mitochondrial and synaptic function in an advanced age mouse model of Alzheimer’s disease

**DOI:** 10.1111/acel.13368

**Published:** 2021-05-05

**Authors:** Fang Du, Qing Yu, Shijun Yan, Zhihua Zhang, Jhansi Rani Vangavaragu, Doris Chen, Shi Fang Yan, Shirley ShiDu Yan

**Affiliations:** ^1^ Department of Surgery Columbia University New York NY USA; ^2^ Department of Pharmacology and Toxicology and Higuchi bioscience Center University of Kansas Lawrence KS USA; ^3^ Department of Molecular Pharmacology & Therapeutics Columbia University New York NY USA

**Keywords:** amyloid pathology, mitochondrial Aβ clearance, mitochondrial proteolysis, mitochondria‐related proinflammation, synaptic rescue

## Abstract

Mitochondrial dysfunction is one of the early pathological features of Alzheimer's disease (AD). Accumulation of cerebral and mitochondrial Aβ links to mitochondrial and synaptic toxicity. We have previously demonstrated the mechanism by which presequence peptidase (PITRM1)‐mediated clearance of mitochondrial Aβ contributes to mitochondrial and cerebral amyloid pathology and mitochondrial and synaptic stress in adult transgenic AD mice overexpressing Aβ up to 12 months old. Here, we investigate the effect of PITRM1 in an advanced age AD mouse model (up to 19–24 months) to address the fundamental unexplored question of whether restoration/gain of PITRM1 function protects against mitochondrial and synaptic dysfunction associated with Aβ accumulation and whether this protection is maintained even at later ages featuring profound amyloid pathology and synaptic failure. Using newly developed aged *PITRM1*/Aβ‐producing AD mice, we first uncovered reduction in PITRM1 expression in AD‐affected cortex of AD mice at 19–24 months of age. Increasing neuronal PITRM1 activity/expression re‐established mitochondrial respiration, suppressed reactive oxygen species, improved synaptic function, and reduced loss of synapses even at advanced ages (up to 19–24 months). Notably, loss of PITRM1 proteolytic activity resulted in Aβ accumulation and failure to rescue mitochondrial and synaptic function, suggesting that PITRM1 activity is required for the degradation and clearance of mitochondrial Aβ and Aβ deposition. These data indicate that augmenting PITRM1 function results in persistent life‐long protection against Aβ toxicity in an AD mouse model. Therefore, augmenting PITRM1 function may enhance Aβ clearance in mitochondria, thereby maintaining mitochondrial integrity and ultimately slowing the progression of AD.

## Graphical abstract

Restoration/gain of neuronal PITRM1 function by enhancing its proteolytic activity in the aged AD mouse model significantly promotes synaptic mitochondrial and cerebral Aβ degradation and clearance, and thereby improves mitochondrial and synaptic functions, and alleviates loss of synapses.

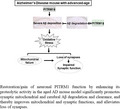

**Abbreviations**
ACSF: artificial cerebrospinal fluid
AD: Alzheimer's disease
APP: amyloid precursor protein
BST: Basal synaptic transmission
Drp1: Dynamin‐related protein 1
EPR: election paramagnetic resonance
GFAP: Glial Fibrillary Acidic Protein
IDE: insulin‐degrading enzyme
LAMP1: Lysosomal‐associated membrane protein 1
LTP: long‐term potentiation
Mfn2: mitofusin 2
MWM: Morris Water Maze
Pitrilysin metallopeptidase 1: PITRM1
presequence peptidase: PreP
RAGE: receptor for advanced glycation end products
TOM: translocase of the outer membrane
WT: wild‐type

## 1 INTRODUCTION

Alzheimer's disease (AD), the most common form of dementia among older people, is a progressive neurodegenerative disease of the brain leading to the irreversible loss of neurons and intellectual abilities, including learning and memory, which become severe enough to impede social or occupational functioning. Extracellular aggregation of Aβ containing senile plaques and intracellular phosphorylated tau protein have long been implicated in progression of AD (Hashimoto et al., 2003; Lustbader, Cirilli, Lin, Hong Wei, et al., 2004). It was reported that intracellular Aβ precedes occurrence of extracellular Aβ deposits in AD, and there is also Aβ exerting toxicity from within the cell apart from extracellular Aβ deposits. There is mounting evidence of the progressive accumulation of Aβ in mitochondria in brains both of transgenic mice and AD patients (Beck et al., 2016; Caspersen et al., 2005; Du et al., 2010; Fang et al., 2015; Hansson Petersen et al., 2008; Lustbader, Cirilli, Lin, Xu, et al., 2004; Reddy & Beal, 2008; Wang et al., 2016; Wang et al., 2020; Yao et al., 2009). Mitochondrial Aβ may bind to many proteins, leading to the sequential events which triggers mitochondrial damage and neuronal cell death (Beck et al., 2016; Du et al., 2008; Lustbader, Cirilli, Lin, Xu, et al., 2004; Yao et al., 2011; Yao et al., 2007). In addition, AD is also associated with synaptic abnormality including synaptic loss (Doherty et al., 2013) and synaptic mitochondria dysfunction (Du et al., 2008, 2010; Yu et al., 2018). Thus, mitochondrial Aβ serves as focal point for potentiating oxidative stress, impairing mitochondrial respiration and energy metabolism, disrupting calcium homeostasis, and altering the balance of mitochondrial dynamics.

Pitrilysin metallopeptidase 1 (PITRM1) (also known as presequence peptidase, PreP), as an M16 metalloprotease containing an inverted zinc‐binding motif and belonging to the pitrilysin oligo peptidase subfamily, is a 117 kDa mitochondrial matrix enzyme responsible for degrading mitochondrial transit peptides after their cleavage, also degrading other unstructured peptides in the range of 10 to 65 residues including the degradation of Aβ (Falkevall et al., 2006; Pinho et al., 2014). Interestingly, PITRM1 is an organellar functional analogue of the human insulin‐degrading enzyme (IDE) which is implicated in AD for the degradation of Aβ (Tanzi et al., 2004). PITRM1 consists of 1037 amino acids residues located on chromosome 10. Although PITRM1 and IDE belong to distinct M16 families, in contrast to IDE, PITRM1 does not degrade insulin. This fact makes PITRM1 a better candidate than IDE for clearing up Aβ. In an ATP independent manner, PITRM1 can completely degrade Aβ40 and Aβ42 as well as the Arctic Aβ40 (E22G) that is associated with increased early onset protofibril formation in Familial AD caused by a mutation of APP gene. Furthermore, in situ, immunoinactivation of PITRM1 in human brain mitochondria revealed complete inhibition of the proteolytic activity against Aβ, proving that under circumstances when Aβ is present in the mitochondria, PITRM1 is the protease responsible for degradation of this toxic Aβ peptides (Alikhani et al., 2009; Falkevall et al., 2006). Decreased PITRM1 proteolytic activity contributes to Aβ accumulation in mitochondria of AD‐affected brain associated with elevated ROS production from dysfunctional mitochondria (Alikhani et al., 2011). Human *PITRM1* mutation (R183Q or T931 M) shows progressive mitochondria and neurodegeneration, including lower mitochondrial oxygen consumption and cytochrome content, reduced capacity to degrade Aβ, and behavioral changes (Brunetti et al., 2016; Langer et al., 2018). This provides a novel link between genetic mitochondrial disease and Aβ accumulation. Thus, eliminating/reducing Aβ in mitochondria could contribute to improving mitochondrial function and halting AD progression.

In the present study, we extend our previous studies by investigating the effects of neuronal PITRM1 on amyloid pathology, mitochondrial function, and synaptic plasticity in the aged AD mouse model (up to 19–24 months), age known to be for late stage with profound AD pathology and pathophysiological changes in synapse. The outcomes of the study address a fundamental unexplored question whether restoration/gain PITRM1 function protects against mitochondrial and synaptic dysfunction associated with Aβ accumulation in a newly developed aged AD mouse model.

## 2 METHODS

### 2.1 Generation of transgenic (Tg) mAPP/*Pitrm1* and mAPP/*mPtrm1* mice

Animal studies were carried out with the approval of the Institutional Animal Care and Use Committees of the University of Kansas‐Lawrence and Columbia University in accordance with the National Institutes of Health guidelines for animal care. Transgenic mice with selective overexpression of neuronal human PITRM1 or with an inactive mutant PITRM1 in which the catalytic base Glu78 in the inverted zinc‐binding motif is replaced by Gln (*mPitrm1* mice as control mice) were achieved under the control of Thy‐1 promoter (termed Tg *Pitrm1* or *mPitrm1*). Tg *Pitrm1* and *mPitrm1* mice in C57BL6/J background have been well characterized and used in our previous study (Fang et al., 2015).

To generate double Tg mice overexpressing PITRM1 or mPITRM1 and Aβ (Tg mAPP/*Pitrm1* or mAPP/*mPitrm1*), Tg *Pitrm1* or *mPitrm1* mice were crossed with Tg *mAPP* mice that overexpress a human mutant form of amyloid precursor protein (APP) bearing both the Swedish (K670 N/M671L) and the Indiana (V717F) mutations (APPSwInd, J‐20 line, obtained from Jackson Lab). Both male and female mice at age from 19 to 24 months were used for the described experiments. Tg *Pitrm1*, *mPitrm1*, and mAPP mice were identified as bearing the transgene from analysis of tail DNA based on PCR amplification using primers for *Pitrm1* [5′‐ CCACAGAATCCAAGTCG‐ 3′ (forward) and 5′‐GTGGAAAATGGATACAGAGT‐3'(reverse)] and for APP [5″‐GACAAGTATCTCGAGACACCTGGGGATGAG‐3′ (forward) and 5′‐ AAAGAACTTGTAGGTTGGATTTTCGTAGCC‐3′ (reverse)]. The investigators were blinded to the mouse genotype in performing experiments.

### 2.2 Synaptic mitochondria preparation

Synaptic mitochondria preparation was previously described (Du et al., 2010; Yan et al., 2018). Briefly, the brain samples were placed in a 5 × volume of ice‐cold isolation buffer [225 mM mannitol, 75 mM sucrose, 2 mM K_2_HPO_4_, 0.1% BSA, 5 mM Hepes, 1 mM EGTA (pH 7.2)]. The tissues were homogenized with a Dounce homogenizer (Kontes Glass Co.). The resultant homogenate was centrifuged at 1300 × g for 5 min, and the supernatant was layered on a 3×2‐mL discontinuous gradient of 15%, 23%, and 40% Percoll. The samples were subjected to a centrifugation at 34,000 × g for 8 min. After centrifugation, band 2 (the interface between 15% and 23% containing synaptosomes) and band 3 (the interface between 23% and 40% containing non‐synaptic mitochondria) were removed from the density gradient. The fractions were then resuspended in 20 mL of isolation buffer containing 0.02% digitonin and incubated on ice for 10 min. The suspensions were then centrifuged at 16,500 × g for 15 min. The resulting loose pellets were washed for a second time by centrifugation at 8,000 × g for 10 min. Pellets were collected and resuspended in isolation buffer. A discontinuous Percoll density gradient centrifugation was performed as described above for a second time. After the centrifugation, the third band was obtained and resuspended in isolation buffer to centrifuge at 16,500 × g for 15 min. The resultant pellet was washed in isolation buffer at 8,000 × g for 10 min. The final synaptic mitochondrial pellet is resuspended in isolation buffer and stored on ice. Protein concentration was determined using the Bio‐Rad DC protein assay (Bio‐Rad Laboratories).

### 2.3 Quantification of mitochondrial Aβ level and cerebral amyloid pathology

Aβ Measurement. Synaptic mitochondrial fractions and cortical homogenates were incubated in 5 M guanidine HCl and 50 mM Tris‐HCl (pH 8.0) overnight and then subjected to Aβ detection using human or mouse Aβ1‐40 and Aβ1‐42 ELISA kits (Invitrogen) following the manufacturer's instructions (Du et al., 2017; Fang et al., 2015).

For quantification of cerebral Aβ deposition, anesthetized mice were perfused transcardially with 0.9% sodium chloride for 10 min and then 4% paraformaldehyde for 30 min. The brains were removed, kept in 4% paraformaldehyde at 4°C for 7 days. Brain sections (30 μm thicknesses) were immersed in wash buffer (sodium phosphate 0.1 M, sodium chloride 0.5 M, Triton X‐100, sodium azide) pH 7.4 for 30 min. Block endogenous peroxidase activity with 3% H_2_O_2_ in methanol for 15 min. After a pre‐incubation for 1 h in blocking solution (10% normal goat serum, 0.3% Triton X‐100 in PBS), sections were incubated overnight at 4℃ with primary antibody 3D6 (1:2000, provided by Eli Lilly) followed by anti‐mouse biotin (1:100 for 1 hour) and HRP (1:100 for 1 hour), and finally color developed with AEC. The area of plaque in the cortex from multiple sections at the same level in each experimental group was determined by image analysis using Universal Images software (Universal Imaging Corp).

### 2.4 Cytochrome c oxidase (CcO) activity assay

The cytochrome *c* oxidase activities of mitochondrial fractions were measured with a cytochrome *c* oxidase kit (Sigma). Briefly, a suitable volume of mitochondria fraction or tissues and enzyme solution was added to 950 µl assay buffer. The reaction was initiated by the addition of 50 µl freshly prepared ferrocytochrome c substrate solution into the cuvette. Changes in OD values at 550 nm were recorded immediately using a kinetic program with 5 seconds’ delay, 10 seconds’ interval; a total of six readings were obtained utilizing an Amersham Biosciences Ultrospect 3100 pro spectrophotometer.

### 2.5 Measurement of ATP and ROS levels

ATP levels in brains of Tg mice were determined using an ATP Bioluminesence Assay Kit (Roche) following the manufacturer's instruction. Brain tissues were homogenized in the lysis buffer provided in the kit, incubated on ice for 15 min, and centrifuged at 14,000 g for 15 min. Subsequent supernatants were measured for the ATP levels using Luminescence plate reader (Molecular Devices) with an integration time of 10 seconds.

Evaluation of intracellular ROS levels was accessed by election paramagnetic resonance (EPR) spectroscopy as described in our previous study (Du et al., 2017). CMH (cyclic hydroxylamine 1‐hydroxy‐3‐methoxycarbonyl‐2,2,5,5‐tetramethyl‐pyrrolidine, 100 μM) was incubated with hippocampal slices for 30 min and then washed with cold PBS. The tissues were collected and homogenized with 100 μl of PBS for EPR measurement. The EPR spectra were collected, stored, and analyzed with a Bruker EleXsys 540x‐band EPR spectrometer (Billerica, MA) using the Bruker Software Xepr (Billerica, MA).

### 2.6 Immunoblotting analysis

Mice hippocampal tissues were homogenized in extraction buffer (10 mM Tris‐HCl, pH 7.4, 100 mM sodium chloride, 1 mM EDTA, 1 mM EGTA, 1 mM sodium fluoride, 20 mM sodium pyrophosphate, 2 mM sodium orthovanadate, 1% Triton X‐100, 10% glycerol, 0.1% SDS, 0.5% deoxycholate, 1 mM PMSF) containing protease inhibitor mixture (set V, EDTA‐free; Calbiochem). Equal amounts of protein were loaded and separated by SDS‐PAGE and transferred to nitrocellulose membrane. The membrane was blocked in TBS containing 5% non‐fat dry milk for 1 h at room temperature and then incubated with antibodies: rabbit anti‐PSD‐95 (ab16495, Abcam, 1:3000), rabbit anti‐synapsin 1 (s8067, Sigma, 1:5000), rabbit anti‐PITRM1 (provided by Dr. Elzbieta Glaser), rabbit polyclonal anti‐synaptophysin (MAB5258, Chemicon, 1:5000), mouse anti‐ Complex IV (cytochrome *c* oxidase, COXIV, ab109863, Abcam, 1:1000), rabbit anti‐LAMP1 (Lysosomal‐associated membrane protein 1, C54H11), (3243, Cell Signaling, 1:1000), rabbit anti‐calnexin (2433, Cell Signaling Technology, 1:1000), mouse anti‐HSP60 (Enzo, ADI‐SPA 806‐D, 1:1000), mouse anti‐VDAC (529532, Calbiochem, 1:1000), rabbit anti‐Mfn2 (WH0009927 M3, Sigma, 1:1000), rabbit anti‐phospho‐Drp1(Dynamin‐related protein 1, Ser616, 3455 s, cell signaling, 1:1000) and mouse anti‐Drp1 (611113, BD Science, 1:3000) overnight at 4°C followed by incubation of secondary antibody for 1 h. After chemiluminescence analysis, the membrane was stripped and reprobed with mouse anti‐β actin (1:10,000, A5441, Sigma). ImageJ software (National Institutes of Health) was used for quantification of intensity of the immunoreactive bands in the scanned blots.

### 2.7 Immunofluorescent staining

Brain slices from the indicated Tg mice were co‐immunostained with primary antibodies: rabbit anti‐PITRM1 IgG antibody (1:2000), mouse anti‐Cytochrome *C* Oxidase (CCO, 1: 5000), PSD‐95 (ab16495, Abcam, 1:3000), synapsin 1 (s8067, sigma 1:5000), mouse anti‐MAP2 (1:5000, sc‐33796, Santa Cruz Biotechnology), rat anti‐CD11b (550282, BD Pharmingen, 1:1000), and mouse anti‐GFAP (Glial Fibrillary Acidic Protein, 3670, Cell Signaling, 1:3000) at 4°C overnight. Sections were then incubated with Alexa Fluor 488‐conjugated goat anti‐rabbit IgG/ 594 goat anti‐mouse or rat IgG or Alexa Fluor 594‐conjugated goat anti‐rabbit IgG/488 goat anti‐mouse IgG secondary antibodies, respectively, for 1 h at room temperature. Nuclei were stained by DRAQ5 (5 μM, Cell Signaling) for 10 min at room temperature. Images were taken using a Leica LS5 Confocal Microscope and analyzed using Universal Metamorph Image Program.

### 2.8 Real‐time PCR

Total RNA was extracted from hippocampus of Tg mice using RNeasy Mini Kit (Cat#: 74104, QIAGEN) according to the manufacturer's protocol. 1ug RNA was proceed directly to produce cDNA using high‐Capacity cDNA reverse transcription kit (Cat#: 4368814, Thermo Fisher). Real‐time PCR was utilized for quantification of gene expression of inflammatory mediators: IL‐1β (Mm00434228‐m1, Thermo Fisher) and TNF‐α (Mm99999068‐m1, Thermo Fisher) and microglial markers: CD11b (Mm00434455‐m1, Thermo Fisher) and CD4 (Mm00442754‐m1, Thermo Fisher). Quantitative real‐time PCR was performed using QuantStudio^TM^ 7 Flex Real‐Time PCR system (Thermo Fisher). Data are calculated using the 2 ^−ΔΔ^ *^Ct^* method as described by the manufacturer and are presented as the fold‐change in gene expression normalized to the indicated endogenous reference genes and relative to the control.

### 2.9 Electrophysiological studies

Electrophysiological recordings were performed as described (Yu et al., 2018). Transverse hippocampal slices (400 µm in thickness) were cut from the Tg mouse brain and maintained in an interface chamber at 29°C and perfused with artificial cerebrospinal fluid (ACSF) continuously bubbled with 95% O_2_ and 5% CO_2_. The ACSF composition in mM was 124 mM NaCl, 4.4 mM KCl, 1 mM Na_2_HPO_4_, 25 mM NaHCO_3_, 2 mM CaCl_2_, 2 mM MgSO_4_, and 10 mM glucose. Field‐excitatory postsynaptic potentials (fEPSPs) were recorded from the CA1 region of the hippocampus by placing the stimulating electrode at the level of the Schaeffer collateral fibers, whereas the recording electrode was placed in the CA1 stratum radiatum. Extracellular responses were acquired using Clampex Software 10.2 (Molecular Device) and a microamplifier (IE‐210, Warner Instruments). Basal synaptic transmission (BST) was assayed by plotting of the stimulus voltage (V) against slopes of fEPSP to generate input–output relations. For LTP experiments, a test pulse was applied every minute at an intensity that evokes a response~35% of the maximum evoked response. LTP was induced by θ‐burst stimulation (4 pulses at 100 Hz, with the bursts repeated at 5 Hz and each tetanus including 3 ten‐burst trains separated by 15 sec).

### 2.10 Behavioral test

Investigators were unaware of mouse genotypes until the behavioral tests were finished. The Morris Water Maze (MWM) test was performed according to the published method (Vorhees & Williams, 2006; Yu et al., 2018). The apparatus mainly composes a pool, which is 150 cm in diameter and 50 cm in height, a platform placed in one of the fixed quadrants for mice to escape, and a camera above the center of the pool to capture the images of the swimming mice. The tank was filled with water kept at 23 ± 2°C during the trials. The platform was hidden 0.5–1 cm below the water surface, and the white paint was used to better cover the platform. In spatial acquisition session, mice were trained for 7 consecutive days with four trials each mouse per day. A trial started with releasing one mouse facing the pool wall, and the mouse was allowed to swim freely and search for the escape platform. If the mouse could not reach the platform within 60 s, it was guided to the platform and allowed to stay on for 15 s before the next trial. The escape latency was analyzed by the behavior software system (HVS water 2020). On the day 8, a probe trial was performed to assess the spatial memory of mice. The platform was removed from the pool, and the mice were allowed to swim freely for 60 s. Traces of mice were recorded, and data were analyzed by HVS water 2020.

### 2.11 Statistical analysis

Statistical analysis was performed using Statview statistics software. Differences among means were assessed by one‐way analysis of variance (ANOVA) followed by Fisher's protected least significant difference for post hoc comparisons. *p* < 0.05 was considered significant. All data were expressed as the mean ± SEM.

## 3 RESULTS

### 3.1 Expression of neuronal PITRM1 in *Pitrm1* and mAPP mice

**FIGURE 1:**
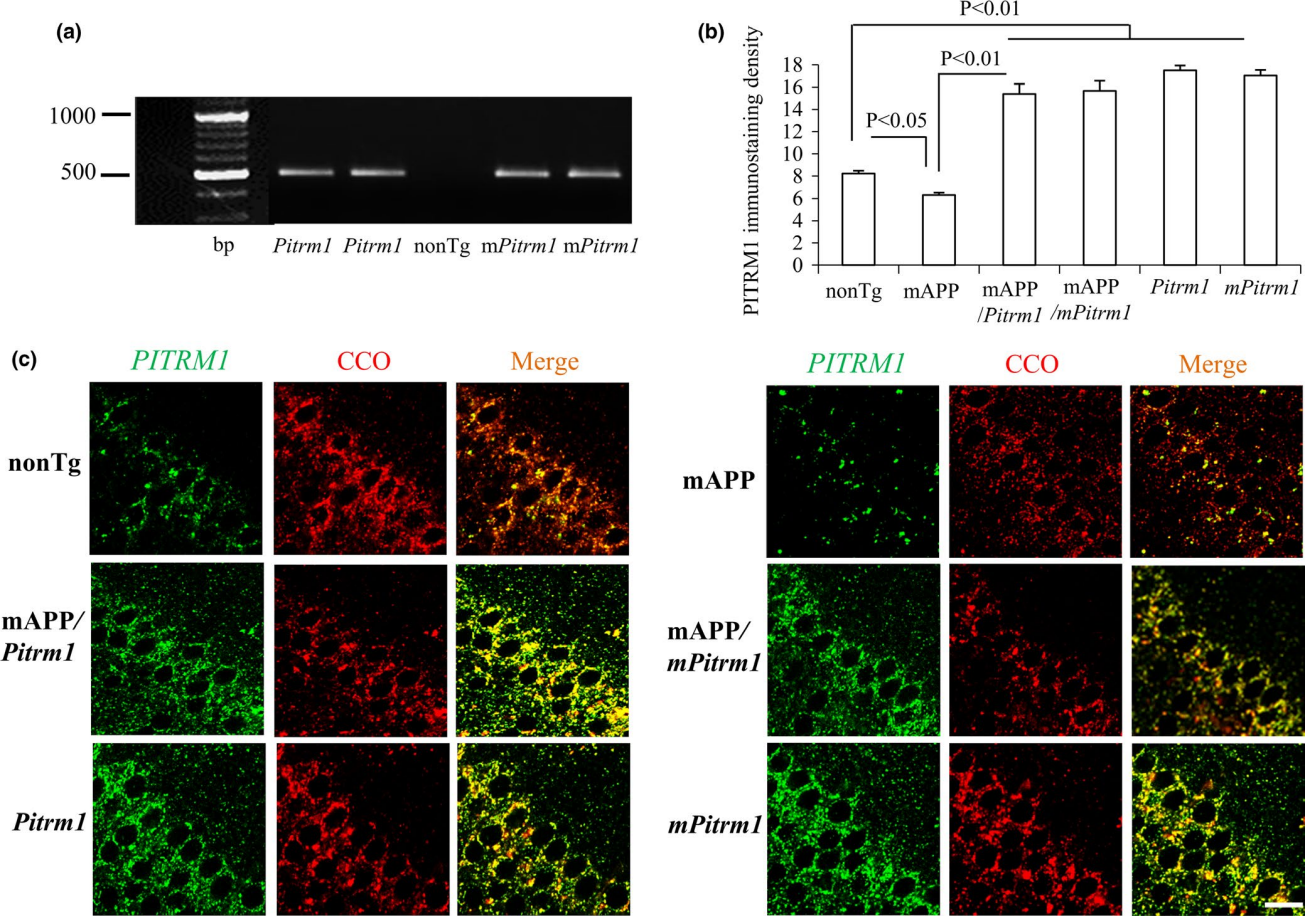
Characterization of transgenic mice. (a) PCR for identification of transgenic *Pitrm1* and *mPitrm1* mice. Tg *Prtirm1* or *mPitrm1* mice were identified as bearing the transgene from analysis of tail DNA based on PCR amplification. (b, c) Brain hippocampal slices from indicated Tg mice were subjected to confocal microscopy with double immunofluorescent staining of PITRM1 (Green) and the mitochondrial marker, CCO (Cytochrome *C* oxidase, Red), to show the mitochondrial localization of PITRM1. Quantifications of the intensity of PITRM1 immunofluorescent staining in (b), and representative staining image of PITRM1 (Green), CCO (Red), and merge with PITRM1 with CCO (yellow) in (c). Scale bar =20 µm. *N* = 3 mice (1–2 male and 1–2 female per group) at age of 19–21 months old

Transgenic *Pitrm1* or *mPitrm1* mice overexpressing PITRM1 or defective PITRM1 activity, as previously described (Fang et al., 2015), were identified by PCR with specific PITRM1 primers (Figure 1a). To determine the possible effect of age on PITRM1 alterations, we performed double immunofluorescence staining of brain sections with PITRM1 and cytochrome *c* oxidase (mitochondrial marker) under confocal microscope to verify neuronal PITRM1 expression and mitochondrial localization. By quantification of immunostaining intensity, PITRM1 expression was increased in cortical neurons in *Pitrm1*, *mPitrm1*, mAPP/*Pitrm1*, and mAPP/*mPitrm1* mice compared to nonTg mice. PITRM1 expression levels were comparable between *Pitrm1* and *mPitrm1* mice (Figure 1b,c). Both staining images were identically overlaid (yellow) by PITRM1 (green, Figure 1c) and CCO (red, Figure 1c). These data verify that PITRM1 protein is correctly targeted to neuronal mitochondria in PITRM1‐expressed mAPP mice. PITRM1 is characterized of neuronal expression by double staining of PITRM1 (green) with MAP2 (I, neural marker). Elevated PITRM1 expression was not found in CD11b‐positive microglia (II, microglial marker) or GFAP‐positive astrocytes (III, astrocytic marker) in the hippocampal slices from the *Pitrm1* mouse (Figure S1). Additionally, when compared to nonTg mice, PITRM1 expression significantly reduced in old mAPP hippocampal neurons (Figure 1b,c), suggesting negative effects of age‐induced high levels Aβ on PITRM1 expression.

### 3.2 Aβ levels in synaptic mitochondria and brain of TG mice

**FIGURE 2:**
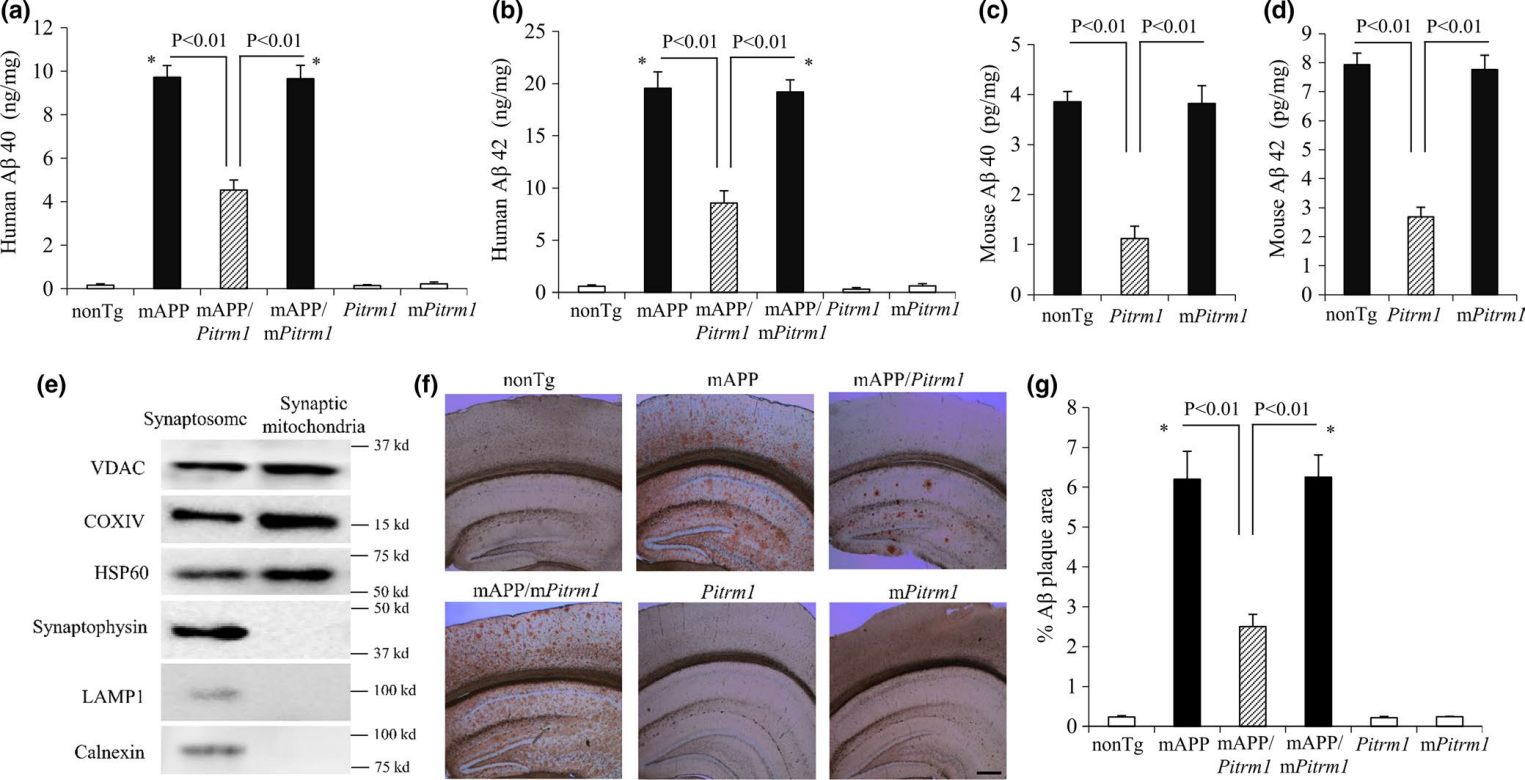
Effect of PITRM1 overexpression on mitochondrial and cerebral Aβ load in aged mAPP mice (a–d). Aβ levels in the synaptic mitochondrial fractions from the hippocampus in the indicated Tg mice were measured by ELISA. Human (a, b) and mouse Aβ levels (c, d) in the synaptic mitochondrial fractions from the indicated Tg mice. *p < 0.01, versus nonTg, Tg Pitram1 and mPitirm1 mice. (e) Characterization of isolated synaptic mitochondrial fractions. To verify the preparation of synaptic mitochondrial fractions, synaptosomal and synaptic mitochondrial fractions were subjected to immunoblotting with antibodies specific to synaptophysin (synaptic protein marker), LAMP1 (lysosome marker), and Calnexin (endoplasmic reticulum marker); VDAC mitochondrial outer membrane protein; COXIV (mitochondrial inner membrane protein) and HSP60 (mitochondrial matrix protein). (f, g). Immunohistochemical staining to show the effect of PITRM1 on Aβ deposition in 19–21 months old mAPP mice. Representative sections stained with Aβ antibody in the indicated Tg mice at 19–21 months of age (f). Quantification of Aβ‐immunoreactive plaques in cerebral cortex including hippocampus in the indicated Tg mice. Data were expressed by the percentage of area occupied by Aβ‐positive plaque (g). No Aβ plaque was found in nonTg, Tg *Pitrm1*, and *mPitrm*1 mice. *N* = 5–6 mice (1–5 male and 1–5 female per group). Scar bar = 30 µm

Next, we examined the effect of PITRM1 overexpression on mitochondrial Aβ accumulation and amyloid pathology in aged mAPP brain. The synaptic mitochondrial pool of human Aβ40 and Aβ42 was significantly reduced in mAPP/*Pitrm1* mice but not in mAPP/*mPitrm1* mice when compared to mAPP mice (Figure 2a,b). We have previously demonstrated age‐dependent decreased PITRMI proteolytic activity in wild‐type (WT) and transgenic mAPP mice expressing Aβ, when comparing the activity of PITRM1 in five‐month and twelve‐month‐old WT and Tg mAPP mice (Alikhani et al., 2011). Decreased PITRMI activity correlates with mitochondrial Aβ accumulation. We thereby evaluated the effect of age on naturally produced endogenous mouse Aβ. Mouse endogenous Aβ was greatly reduced in *Pitrm1* mice but not in *mPitrm1* mice (Figure 2c,d). The purity of synaptic mitochondria was confirmed by the enrichment of mitochondrial markers (VDAC, cytochrome *c* oxidase/COXIV and HSP60), but absence in synaptophysin, lysosomal (LAMP1), and endoplasmic reticulum (Calnexin) markers (Figure 2e).

To determine whether PITRM1 alters amyloid pathology, we quantified Aβ‐positive deposition by immunostaining of brain sections with specific Aβ antibody. MAPP mice revealed significant amount of Aβ‐positive deposition as compared to nonTg mice (Figure 2f,g), whereas mAPP/*Pitrm1* but not mAPP/*mPitrm1* mice significantly reduced Aβ deposits (Figure 2f,g). These data indicate that increased PITRM1 activity/expression largely reduces mitochondrial and cerebral Aβ accumulation in the aging brain and Aβ‐expressing AD mice. Loss of PITRM1 activity results in Aβ accumulation.

### 3.3 PITRM1 rescues synaptic mitochondrial function and inhibits inflammation in aged MAPP/*Pitrm1* mice

**FIGURE 3:**
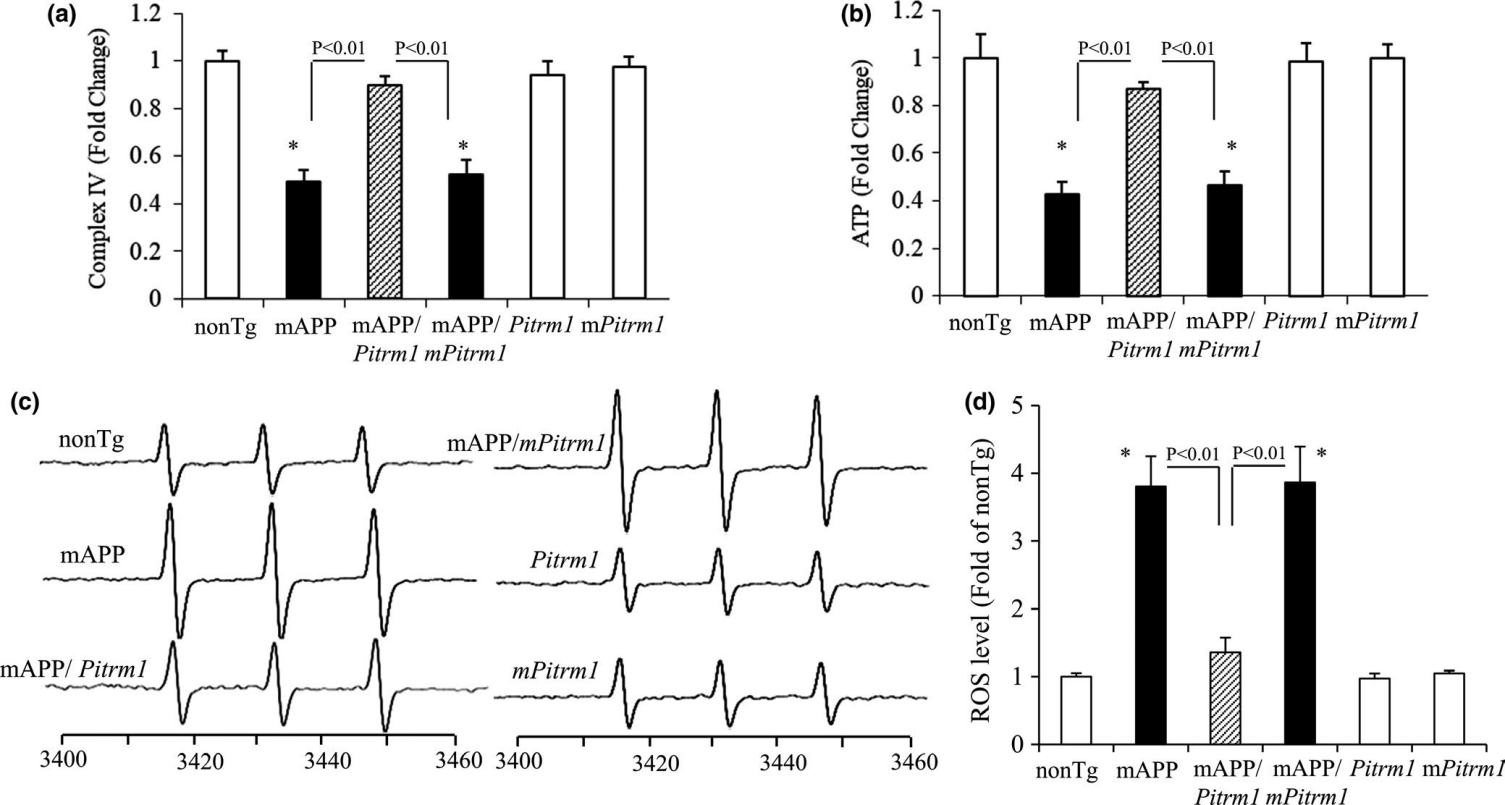
Effect of PITRM1 overexpression on mitochondrial function in aged mAPP mice. Complex IV/cytochrome *c* oxidase (CCO) activity (a), ATP (b), and ROS levels (c, d) in brain hippocampal tissue of the indicated 19–21 months old Tg mice were measured. Representative EPR spectra (c) and quantification (d) for ROS signals in the indicated groups. *p < 0.01, versus nonTg, Tg *Pitrm1*, and *mPitrm1* mice. *N* = 3 mice (1–2 male and 1–3 female per group)

To determine whether reducing mitochondrial Aβ improves mitochondrial function in aged AD mice, we analyzed mitochondrial respiratory function by measuring activity of cytochrome c oxidase, a key enzyme in the terminal step of mitochondrial electron transport chain (complex IV), and energy metabolism by ATP level. The activity of complex IV was significantly decreased by ~40% in mAPP cortical mitochondria as compared with those in nonTg mitochondria (Figure 3a). In contrast, mAPP/*Pitrm1* mitochondria restored activity of complex IV when compared to mAPP mitochondria (Figure 3a). Similarly, mAPP/*Pitrm1* mitochondria had a higher level of ATP than mAPP mitochondria (Figure 3b). There was no difference of complex IV activity and ATP level between mAPP and mAPP/*mPitrm1* mice (Figure 3a,b).

Because mitochondria are a major resource of ROS generation, we assessed whether increased PITRM1 expression attenuates ROS production in Aβ‐affected brain. Consistent with our previous results (Du et al., 2017; Du et al., 2008; Fang et al., 2015), brains of old mAPP mice demonstrated remarkably increased intracellular ROS levels by ~fourfold using a highly specific EPR spectroscopy in hippocampus. ROS were largely abolished in mAPP/*Pitrm1* mice but not in mAPP/*mPitrm1* mice (Figure 3c,d).

**FIGURE 4:**
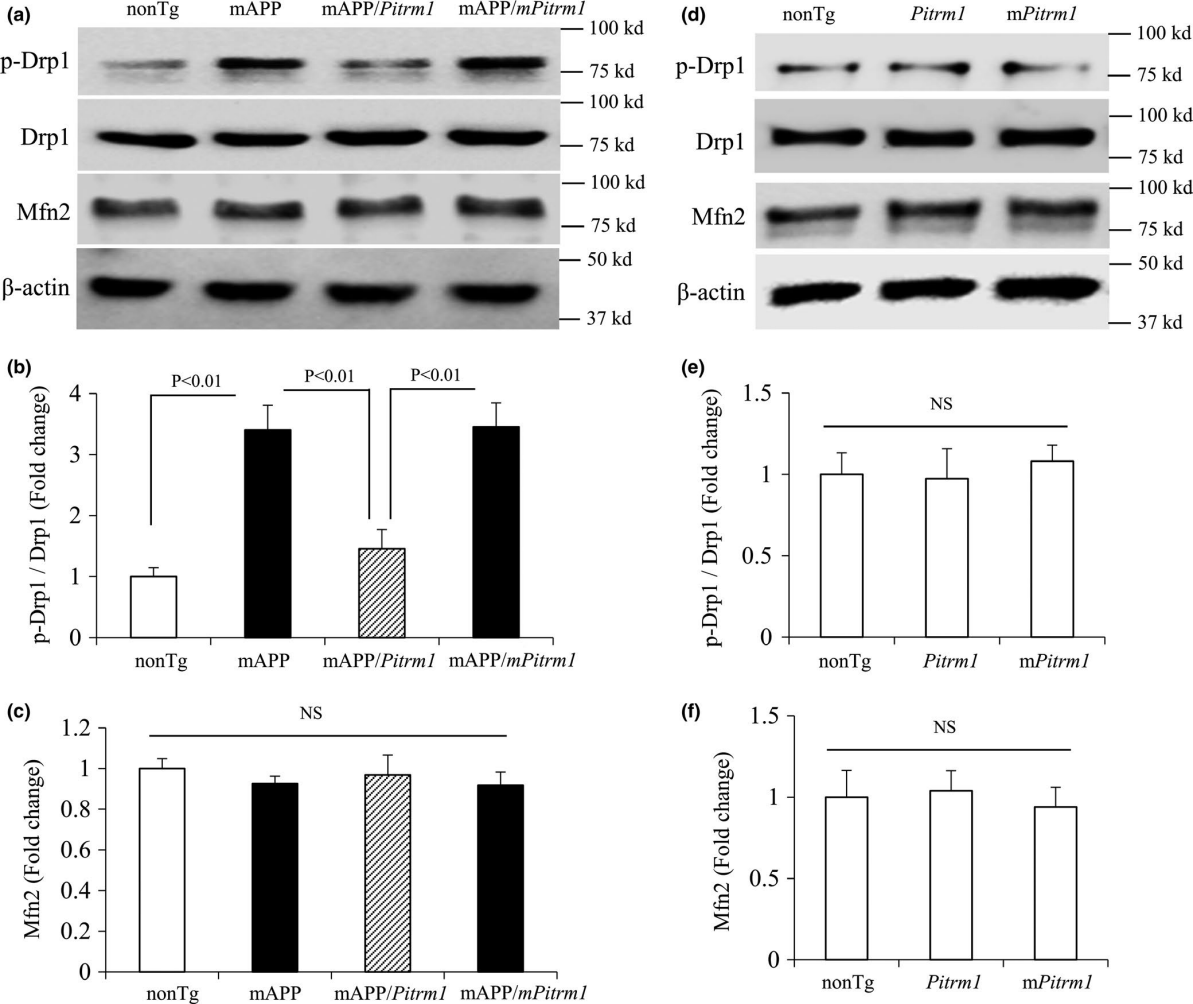
Effect of PITRM1 overexpression on mitochondrial Drp1 expression in aged mAPP mice. Immunoblotting of hippocampal homogenates of the indicated 19–21 months old mice. Representative immunoblots show phosphorylation of Drp1 (p‐Drp1), total Drp1, MFN‐2, and β‐actin (a, d). β‐actin is used for protein loading control. b, c and e, f denote densitometry of immunoreactive bands of the indicated Tg mice. *N* = 3 (1–2 male and 1–2 female per group)

We next investigated whether PITRM1 overexpression altered levels of mitochondrial fission and fusion proteins in aged AD mice brains (Figure 4). An increase in Ser616 phosphorylated Drp1 expression, which is critical for controlling mitochondrial fission [16], was observed in hippocampus of the mAPP and mAPP/*mPitrm1* mice at 19–21 months old as compared to nonTg mice (Figure 4a,b). However, compared to mAPP mice, mAPP/*Pitrm1* mice significantly blocked elevation of phosphorylated Drp1 (Figure 4a,b). In contrast, Mfn2 (mitofusin 2), a mitochondrial membrane protein that participates in mitochondrial fusion, has no changes in mAPP mice compared to nonTg mice (Figure 4a,c). It was noted that levels of phosphorylated Drp1 or MFN2 were comparable among *Pitrm1*, *mPitrm1*, and nonTg mice, indicating no effect of PITRM1 overexpression on mitochondrial fission and fusion protein expression in the absence of Aβ (Figure 4d–f). These results suggest that PITRM1 overexpression effectively reduced excessive mitochondrial fission in aged AD mice brains enriched for Aβ.

**FIGURE 5:**
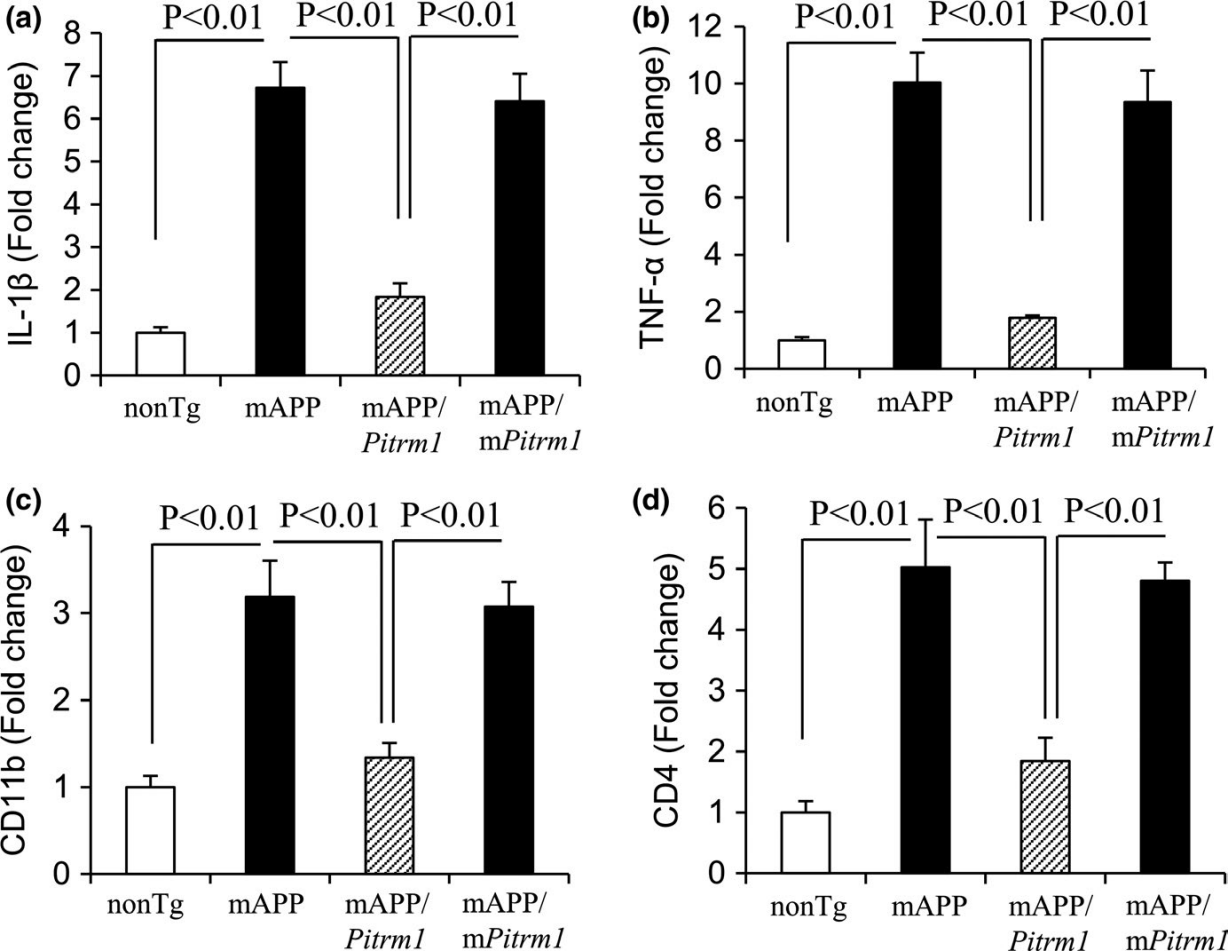
Effect of PITRM1 overexpression on induction of proinflammatory cytokines and microglial activation in aged mAPP mice hippocampi. Quantitative real‐time PCR analysis of cytokines (IL‐1β, a and TNF‐α, b;) and microglial makers (CD11b, c and CD4, d) of total RNA extracted from hippocampus of the indicated Tg mice at 19–20 months of age. *N* = 4 mice (1–2 male and 2–3 female per group)

Given that mitochondria function as signaling platforms in the proinflammatory response by activation of proinflammtory mediators (Krysko et al., 2011), we hypothesized that protective effect of PITRM1 on mitochondrial function may lessen inflammation in aged AD brain. Consistent with our previous studies (Fang et al., 2015), increased PITRM1 expression effectively attenuated up‐regulated production of proinflammatory cytokines (*IL*‐*1β* and *TNF*‐*α*, Figure 5a,b) and suppressed of microglial activation markers (*CD11b* and *CD4*, Figure 5c,d) in aged mAPP hippocampi. Together, these data suggest that increased PITRM1 expression/activity suppresses ROS production, inhibits inflammation, and restores mitochondrial respiratory function and bioenergy.

### 3.4 Effect of PITRM1 overexpression on synaptic and cognitive function and synapses in mAPP mice

**FIGURE 6:**
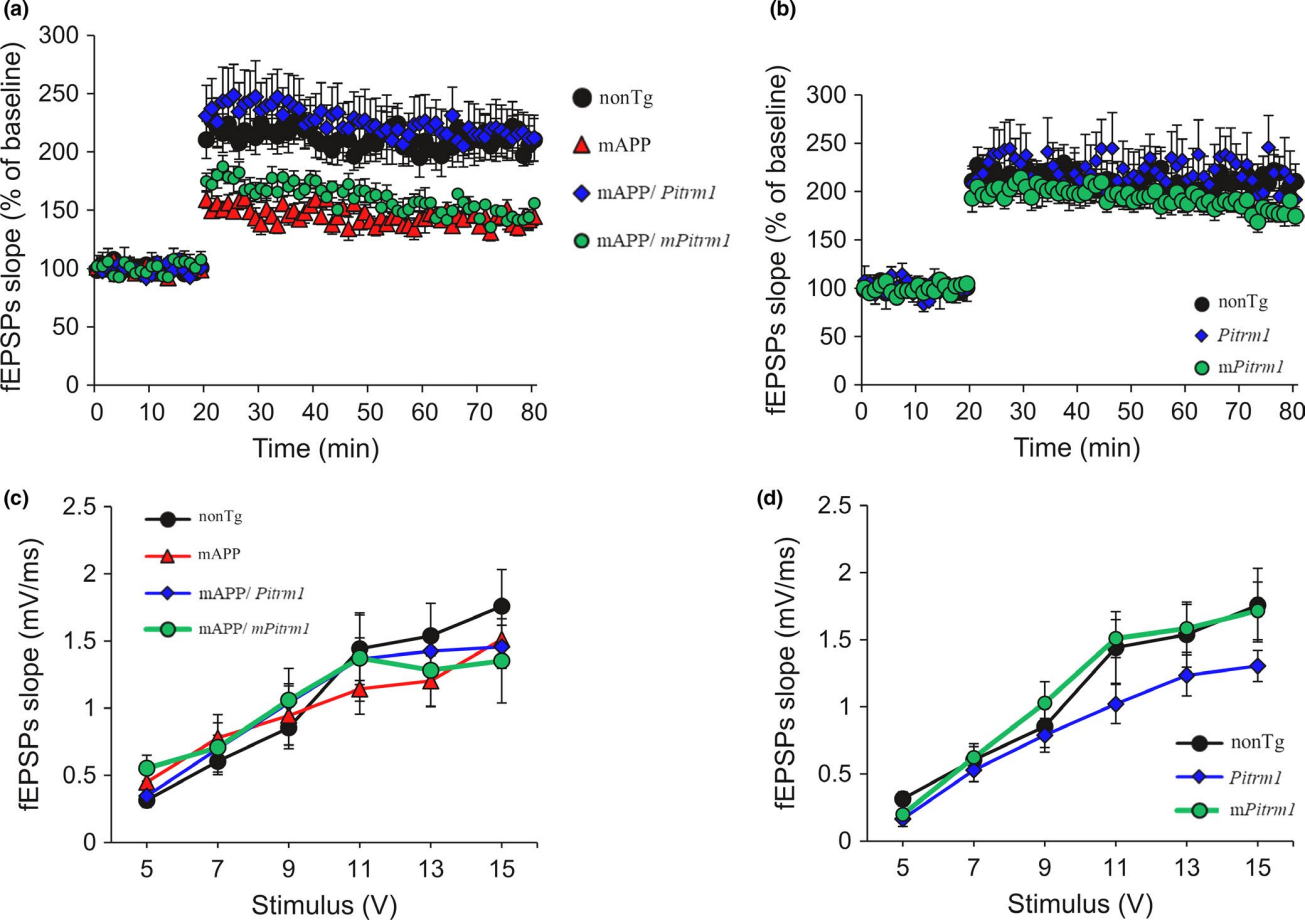
Effect of PITRM1 overexpression on long‐term potentiation (LTP) impairment in aged mAPP mice. LTP (a.b) and basal synaptic transmission,fEPSP (c.d), were recorded in hippocampal slices of nonTg (nine slices from three female mice), mAPP (8 slices from three female mice), mAPP/*Pitrm1* (12 slices from two males and two females), and mAPP/*mPitrm1* (nine slices from three male mice) from 19 to 24 months of age. *N* = 3–4 mice (two male and 2–3 female per group)

Given the importance of synaptic mitochondria in the maintenance of synaptic and cognitive functions, we examined synaptic transmission under basal conditions and during long‐term potentiation (LTP), a form of synaptic plasticity that is widely studied as a cellular model for learning and memory. In a previous study, we demonstrated the protective effect of PITRM1 overexpression on LTP in mAPP/*Pitrm1* mice in comparison with those in mAPP mice at 12 months of age (Fang et al., 2015). Here, we assessed synaptic plasticity in the aged mice to determine whether LTP was also improved in the aged mAPP/*Pitrm1* mice by recording CA1 neurons in hippocampal slices. Consistent with our previous study, LTP was significantly decline in mAPP mice, whereas mAPP/*Pitrm1* mice showed a largely restored LTP, but no such protection was found in mAPP/*mPitrm1* mice (Figure 6a). There were no significant differences of the basal neurotransmission (field‐excitatory postsynaptic potential, fEPSPs) and LTP in the CA1 stratum radiatum between *Pitrm1*, *mPitrm1*, and nonTg mice (Figure 6b–d), indicating no effect of PITRM1 overexpression on synaptic function.

**FIGURE 7:**
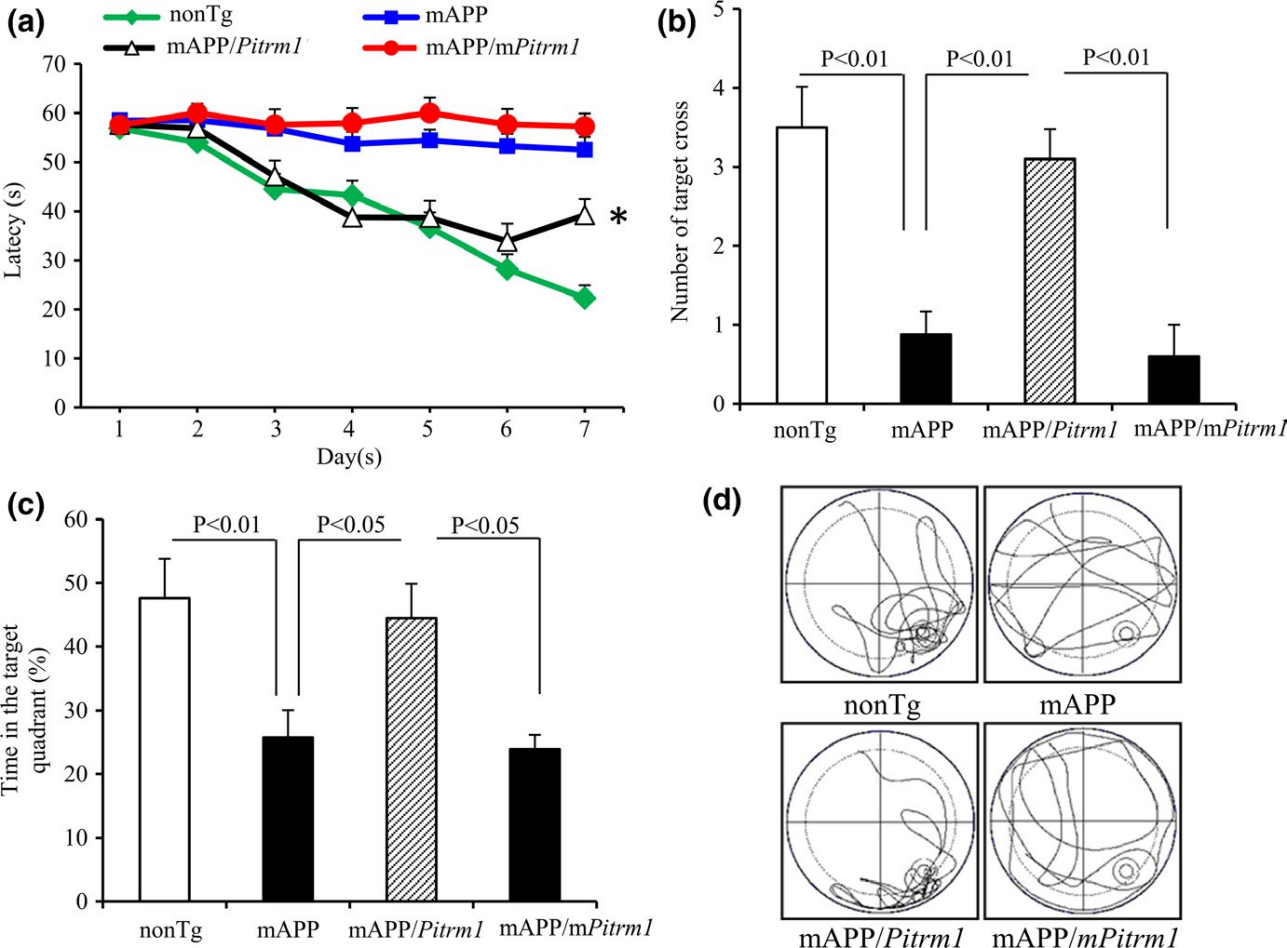
Effect of PITRM1 overexpression on learning memory deficiency in aged mAPP mice. (a–c). Results of the Morris Water Maze test showed the average latency to escape to locate the hidden platform during each day of training sessions (a), *p < 0.01 compared to Tg mAPP or Tg mAPP/*mPitrm1* mice, the mean number of mice crossing the target during probe trials (b), and time spent in the quadrant where the hidden platform is located (c). Tg mAPP and mAPP/*mPitrm1* mice showed much less preference for the target quadrant compared to nonTg mice. MAPP/ *Pitrm1* mice demonstrated an increase in the percentage of time spent in the target quadrant compared to mAPP or mAPP/*mPitrm1* mice. (d) Pattern of representative searching traces for the indicated Tg mice in search of the target. *N* = 5–10 mice (3–6 male and 1–7 female per group) at age of 19–21 months

We further examined whether increased PITRM1 expression improves cognitive function using a Morris Water Maze (MWM) test. Compared to mAPP mice, mAPP/*Pitrm1*, but not mAPP/*mPitrm1*, mice showed significantly improved spatial reference memory with a shorter latency to locate the hidden platform during the training session (Figure 7a). Similarly, mAPP/*Pitrm1* mice revealed increased the number of times crossing the target (Figure 7b,d) and time in the target quadrant (Figure 7c,d) in the recording period. Mice among tested groups had similar swimming speed by the visual swimming speed test (Figure S2). These results demonstrate that mAPP/*Pitrm1* retained a better searching strategy. These data indicate that increased neuronal PITRM1 expression and activity improves learning and memory in mAPP mice.

**FIGURE 8:**
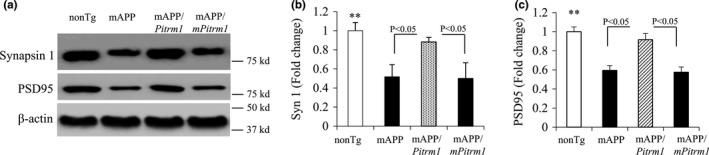
Effect of PITRM1 on synaptic protein expression in aged mAPP mice. The hippocampal homogenates from the indicated 19–21 months old Tg mice were subjected to immunoblotting to detect synapsin 1, PSD95 (a, upper panel), and β‐actin (a, lower panel). Quantifications of intensity of immunoreactive band for synapsin 1 (b) and PSD95 (c) are shown in b and c, respectively. *N* = 3 mice (1–2 male and 1–3 female per group). *p < 0.01, versus mAPP and mAPP/*mPitrm1* mice

Finally, we evaluated whether the neuroprotective effect of PITRM1 on synaptic plasticity deficits reflects synaptic morphology by examining expression levels and densities of two important synaptic proteins (synaptic vesicles protein‐Synapsin 1 and postsynaptic density protein 95‐PSD95) in mAPP/*Pitrm1* mice compared to mAPP and nonTg mice. Immunoblotting of hippocampal homogenates showed significantly decreased expression levels of synapsin 1 and PSD95 in mAPP mice by 40–50% as compared to nonTg mice. mAPP/*Pitrm1* mice largely restored levels of these two proteins. There was no such protective effect on mAPP/ *mPitrm1* mice (Figure 8a–c).

**FIGURE 9:**
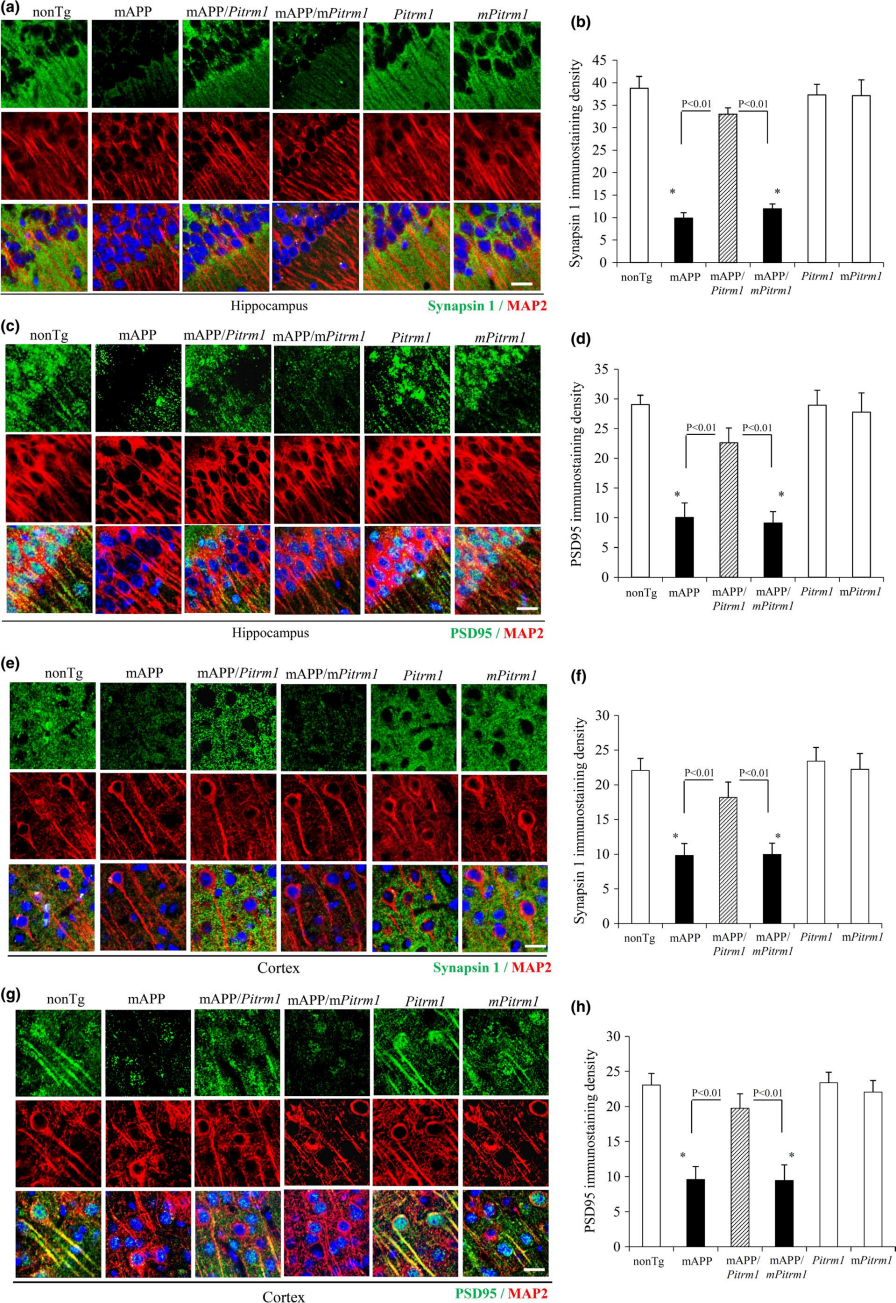
Effect of PITRM1 on synaptic density in aged mAPP mice. Fixed brain slices from the indicated 19–21 months old Tg mice were stained with synapsin 1 (presynaptic marker, a for hippocampus and e for cortex) or PSD95 (postsynaptic marker, c for hippocampus and g for cortex). Nuclei were stained by DRAQ5 as shown in blue. Quantification of the density of synapsin 1‐ (b for hippocampus and f for cortex) and PSD95‐positive clusters (d for hippocampus and h for cortex) was measured in the hippocampus of the indicated Tg mice. *N* = 5–6 mice (1–4 male and 1–5 female per group). Scar bars = 50 µm

To evaluate synaptic density in brains of Tg mice, we performed immunostaining of brain sections with synapsin 1 and PSD‐95 and then quantified by area occupied by these two synaptic proteins. It is clear that synapsin 1 (Figure 9a,b) or PSD‐95‐labeled synaptic densities (Figure 9c,d) were significantly decreased in the hippocampus (Figure 9a–d) and cortex (Figure 9e–h) of mAPP mice as compared to nonTg mice, whereas mAPP/*Pitrm1* mice, but not mAPP/*mPitrm1* mice, robustly restored the synaptic densities of synapsin 1 and PSD‐95. No difference in synaptic densities was found between nonTg, *Pitrm1*, and *mPitrm1* mice (Figure 9a–h). These results demonstrate that enhancing neuronal PITRM1 function attenuates loss of synapses in Aβ‐rich environment.

## 4 DISCUSSION

Mitochondria are central organelle for the regulation of cell survival and death. Neurons are vulnerable to mitochondrial dysfunction due to their high energy demands and dependence on respiration to generate ATP. Mitochondrial dysfunction may, therefore, drive or mediate various AD pathologies. Combing clinical genetic mutation of PITRM1, sporadic AD, and transgenic AD mouse model, progressive accumulation of Aβ in mitochondria is associated with impairment in mitochondrial respiration and ROS production. The formation of mitochondrial Aβ plaques precedes the extracellular Aβ deposition and is evident before the onset of clinical symptom both in AD patients and in transgenic Aβ/AD mice, where there are detected in brain as early as 4 months of age (Caspersen et al., 2005; Du et al., 2010; Lustbader, Cirilli, Lin, Xu, et al., 2004; Manczak et al., 2006). Early mitochondrial Aβ accumulation is consistent with an early onset of loss of synapses and synaptic and mitochondrial damage (Du et al., 2010; Lustbader, Cirilli, Lin, Xu, et al., 2004; Reddy, 2008; Reddy et al., 2012). Thus, accumulation of mitochondrial Aβ may be an initiating or promoting pathological event, leading to mitochondrial and neuronal perturbation.

In our previous studies, we demonstrate that increased neuronal PITRM1 activity attenuated mitochondrial and cerebral amyloid pathology and rescued mitochondrial and synaptic function in Aβ‐producing transgenic AD mice at 12 months of age (Fang et al., 2015). The present study extends our previous findings to address whether the protective effect of PITRM1 overexpression in mAPP mice would keep going to the advanced age (19–24 months). Compared to 12‐month‐old mAPP mice as shown in our previous study​ (Fang et al., 2015), 19–24‐month‐old mAPP mice as a model of late‐stage AD exhibit extensive amyloid pathology, a greater degree of neuronal mitochondrial Aβ accumulation (two‐ and threefold increased Aβ40, 10 ng/mg protein and Aβ42, ~20 ng/mg protein in 19–21 months old mAPP mice vs. Aβ40, ~3.3 ng/mg protein and Aβ42, 10 ng/mg protein in 12‐month‐old mAPP mice), excessive ROS production (fourfold increase vs. 2.2‐fold in 12‐month‐mAPP mice), impaired complex IV activity (~45% reduction in 19–21 months old mAPP mice and ~25% in 12‐month‐old mAPP mice vs. nonTg mice), and severe loss of synapses. These results demonstrate that in an AD mouse model at an advanced age, the progressive development of amyloid pathology and mitochondrial and synaptic defects mirrors those observed in AD‐affected brains.

Age is a risk factor for promoting and amplifying Aβ pathology and synaptic and mitochondrial degeneration. Age‐related oxidative stress may contribute importantly to compromise PITRM1 function as shown by decreased proteolytic activity, thereby reducing the degradation and clearance of mitochondrial Aβ​ (Fang et al., 2015). Thus, it is essential and logical to determine whether constant restoration or increase in PITRM1 expression and function to maintain mitochondrial integrity in older AD mice by degrading and clearing toxic metabolites such as mitochondrial Aβ persistently protects against age‐ and Aβ‐mediated mitochondrial, synaptic, and cognitive perturbation. We observed that 19–21 months old mAPP/*Pitrm1* mice lessened the accumulation of Aβ in synaptic mitochondria and brain. Further, mitochondrial respiratory function and ATP levels were significantly improved in mAPP/*Pitrm1* mice versus mAPP mice. Accordingly, increased PITRM1 alleviated synaptic dysfunction and loss of synapses and improved learn and memory in aged mAPP/*Pitrm1* mice. Therefore, the present results, together with the previous findings, suggest that augmenting PITRM1 function to clear up toxic Aβ from mitochondria gives life‐long persistent protection against Aβ pathology and mitochondrial and synaptic alterations. These studies address fundamental and unexplored questions: (1) Is neuronal PITRM1, in particular proteolytic activity, sufficient to constantly degrade and clear mitochondrial Aβ in the late stage of the AD mouse model even with extensive amyloid pathology and mitochondrial dysfunction? (2) Does restoration of neuronal PITRM1 function also clear age‐related accumulation of toxic metabolite such as naturally produced endogenous Aβ? (3) Does PITRM1‐mediated mitochondrial clearance protect against synaptic perturbation, loss of synapses, and cognitive dysfunction in the late stage of AD? The answers to these questions significantly increase the body of evidence of the contribution of PITRM1 proteolytic activity to mitochondrial quality control and neuronal failure in an Aβ‐rich environment. PITRM1 may be a potential therapeutic target for halting AD progress as well as age‐related dementia and cognitive decline by clearance of accumulation of toxic metabolites in mitochondria and maintenance of mitochondrial integrity.

Aβ is produced by continuous cutting APP in the presence of β‐, γ‐secretase in ER, and released to cytoplasm or extracellular space via Golgi and lysosome/endosome transport pathway. Moreover, extracellular Aβ could transport to organelles through the cellular internalization, receptor for advanced glycation end products (RAGE) (Takuma et al., 2009), the translocase of the outer membrane (TOM) machinery (Hansson Petersen et al., 2008), ER/mitochondrial cross‐talk via mitochondria‐associated membranes (Caspersen et al., 2005; Hedskog et al., 2013), or unknown mechanisms. Aβ may also be locally produced in mitochondria *via* gamma‐secretase that is localized in mitochondria (Behbahani et al., 2010; Pavlov et al., 2011; Teng & Tang, 2005). Mitochondrial isoform of IDE (insulin‐degrading enzyme) or γ‐secretase that can degrade cleaved mitochondrial targeting sequences (Farris et al., 2005; Hansson et al., 2004; Leissring et al., 2004; Pavlov et al., 2011; Teng & Tang, 2005) may be involved in degrading and clearing mitochondrial Aβ. PITRM1 is a novel mitochondrial peptidasome and functions as degradation of Aβ for clearance of mitochondrial Aβ. Indeed, our previous and present studies support that restoring and increasing PITRM1 function reduced mitochondrial pool of Aβ in an AD mouse model. Additionally, increased expression of PITRM1 not only degrades mitochondrial Aβ but also reduces cerebral Aβ deposition, suggesting an important regulating effect of mitochondrial Aβ on the total brain Aβ levels. The localization of overexpression of PITRM1 protein in cortical mitochondria of *Pitrm1* mice (Fang et al., 2015) excludes the possible mis‐localization of PITRM1 from mitochondrial to intracellular/ extracellular compartment. Given that exogenous or intracellular Aβ is capable of direct transport into mitochondria *via* aforementioned pathways, the mitochondrial pool of Aβ may undergo dynamic changes in different intracellular compartments, contributing to the balance of intracellular/extracellular Aβ accumulation. Further investigation is required to elucidate mechanisms underlying regulation of the dynamic changes and balance between mitochondrial and cerebral pool of Aβ.

Functionally, increased expression/activity of PITRM1 are resistant to Aβ‐induced mitochondrial defects as shown by the complete recovery of cytochrome *c* oxidase (complex IV) activity and ATP levels in cortical mitochondria from aged mAPP/*Pitrm1* mice even at the late age of 19–21 months. Mitochondrial respiration is principal function of mitochondria. The respiratory chain reflects electron flux through complexes I, III, and IV, and inactivation of enzyme activities associated with any one of these complexes in the respiratory chain could disrupt the respiratory chain function causing decreased oxygen consumption and respiratory efficiency. Thus, the reduction in the activity of cytochrome *c* oxidase, a component of complex IV of the mitochondrial electron transport chain, could be one of the mechanisms for disturbing mitochondrial respiratory function as shown in mAPP mice.

Given that mitochondrial function is important for maintaining mitochondrial dynamics, augmenting PITRM1 proteolytic function could reverse abnormal mitochondrial fusion and fission balance by suppressing excessive mitochondrial fragmentation in an Aβ rich environment. Indeed, we demonstrated that increased PITRM1 activity completely blocked excessive Drp1 expression, a major protein controlling mitochondrial fragmentation in Aβ‐producing neurons of AD mice. Thus, the detailed involvement of PRITM1 in Aβ‐mediated mitochondrial dynamic alterations requires further investigation in a near future. Additionally, there were no significant changes in autophagy protein LC3‐II, an active form of autophagy, between mAPP and mAPP/*Pitrm1* brains (data not shown), suggesting that PITRM1‐mediated mitochondrial perturbation and Aβ metabolism are unlikely through autophagy pathway. Consistent with previous studies, PITRM1, but not inactive PITRM1, is sufficient to suppress Aβ‐induced the production of proinflammatory mediators and microglial activation in mAPP/*Pitrm1* mice compared to mAPP mice even in older AD mice. This further supports the protective effect of PITRM1 activity on Aβ‐induced inflammation.

Synaptic mitochondria are vital for synaptic function by providing energy, regulation of axonal dendritic development, modulation of calcium signals to power and to regulate synaptic transmission, respectively. Axonal mitochondrial transport is important to maintain synaptic function and synaptic morphology. Thus, dysfunctional mitochondria contribute to synaptic stress. Early synaptic mitochondrial dysfunction associates with Aβ accumulation in synaptic mitochondria and axonal mitochondrial transport (Du et al., 2010). Thus, maintenance of mitochondrial integrity by clearance of accumulation of toxic Aβ in mitochondria are important for synaptic structure and function. Indeed, aged mAPP/*Pitrm1* mice demonstrate an increase in synaptic plasticity and transmission and deceases in loss of synaptic protein expression and synaptic density as compared to mAPP mice. mAPP/*Pitrm1* mice revealed a complete recovery of LTP, increased expression of synaptic proteins (PSD95 and synapsin 1), and restored PSD95‐ or synapsin 1‐positive synapses. Such protective effects of PITRM1 overexpression were not found in mAPP/ *mPitrm1* mice lacking PITRM1 proteolytic activity. Since PSD95 and Synapsin 1 are key proteins related to synaptic plasticity, neuronal development, the release of neurotransmitter, and formation of memories by binding to adhesion molecules (Perdahl et al., 1984), loss of these synaptic proteins could disturb synaptic structure and function. Furthermore, enhancing PITRM1 proteolytic activity greatly improved cognitive function. Therefore, PITRM1 function may be important, at least in part, for the protection against Aβ‐induced alterations in synaptic function and morphology.

In summary, we clearly demonstrate that gaining neuronal human PITRM1 function by enhancing its proteolytic activity in the aged AD mouse model significantly reduces synaptic mitochondrial and cerebral Aβ deposition, improves mitochondrial, synaptic, and cognitive function, and alleviates loss of synapses. Importantly, lack of PITRM1 proteolytic activity fails to afford these protections. These studies significantly increase the body of evidence that PITRM1 functions in clearing and degrading mitochondrial Aβ, contributing to mitochondrial and synaptic pathology in an Aβ‐rich environment. These data support the viewpoint that PITRM1, as a mitochondrial Aβ scavenger, attenuates AD symptoms or halts AD progression even in the late stage with profound amyloid pathology. Therefore, PITRM1 may be a potential therapeutic target for halting AD progress by clearance of mitochondrial toxicity and maintenance of mitochondrial integrity, even at late stage of AD.

## CONFLICT OF INTEREST

We have no conflicts of interest to disclose. We have no contracts related to this research with any organization that could benefit financially from our research.

## AUTHOR CONTRIBUTIONS

S.S.Y. conceived, directed, and developed the concept/project, designed and supervised this study, analyzed/interpreted the results, and wrote manuscript. F. D. conducted immunostaining/ quantification, Aβ measurement and EPR, analyzed data, provided figures, and assisted with the preparation of manuscript. Q.Y. performed RT‐PCR, Western blot, and complex activity and ATP, analyzed results, provided figures, analysis of MWM mice behavior data and provide figure, and assisted with preparation of manuscript. S.Y performed LTP experiments and provided LTP‐related figures. Z.H.Z assisted with preparation of brain sections and Aβ staining. Jhansi Rani Vangavaragu performed MWM mice behavior, analyzed data, and provided figure. D.C maintained and genotyped transgenic mice, and edited manuscript. S.F.Y. provided suggestions and assisted with preparation of manuscript.

## Supporting information

Figures S1–S2

## Data Availability

The data that support the findings of this study are available on request from the corresponding author. The data are not publicly available due to privacy or ethical restrictions.
