# Supplementary material for: Gain of PITRM1 peptidase in cortical neurons affords protection of mitochondrial and synaptic function in an advanced age mouse model of Alzheimer’s disease
Source: Aging Cell. 2021 May 5;20(5):e13368. doi: 10.1111/acel.13368 (PMC8135081; doi:10.1111/acel.13368)
Supplement: Supplementary file 1 — Figures S1–S2 [file ACEL-20-e13368-s001.docx]

**Supporting Information**

**Gain of PITRM1 peptidase in cortical neurons affords protection of mitochondrial and synaptic function in an advanced age mouse model of Alzheimer’s disease**

Fang Du^1#^, Qing Yu^1#^, Shijun Yan^3^, Zhihua Zhang^3^, Jhansi Rani Vangavaragu^3^, Doris Chen^3^, Shi Fang Yan^1^, Shirley ShiDu Yan^1,2^*

^1^Department of Surgery and ^2^Molecular Pharmacology & Therapeutics, Columbia University New York, NY 10032, ^3^ Department of Pharmacology and Toxicology and Higuchi bioscience Center, University of Kansas, KS66047

*Correspondence to:

Shirley ShiDu Yan, M.D

Department of Surgery and Molecular Pharmacology & Therapeutics

Columbia University

E-mail: [sdy1@cumc.columbia.edu](mailto:sdy1@cumc.columbia.edu)

Tel: 1-212-342-0494

^#^ Qing Yu and Fang Du contributed equally to this work.


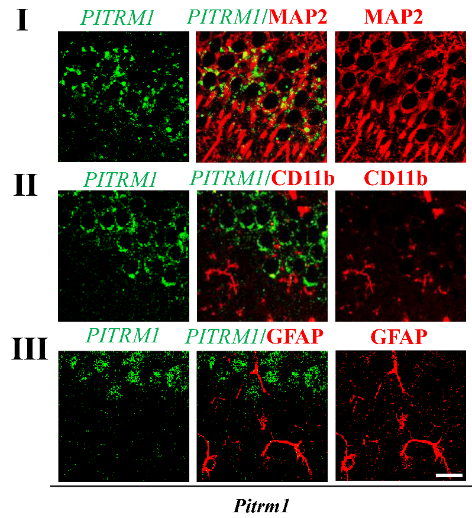


**Supplementary Figure 1.** Characterization of neuronal expression of PITRM1 in mouse brain. Brain slices of hippocampal area from the 19 months old male Tg *Pitrm1* mouse were double-stained with PITRM1 (green) and MAP2 (**I**, neural marker), CD11b (**II,** microglial marker) or GFAP (**III,** astrocytic marker). Scar bar = 50 µm.


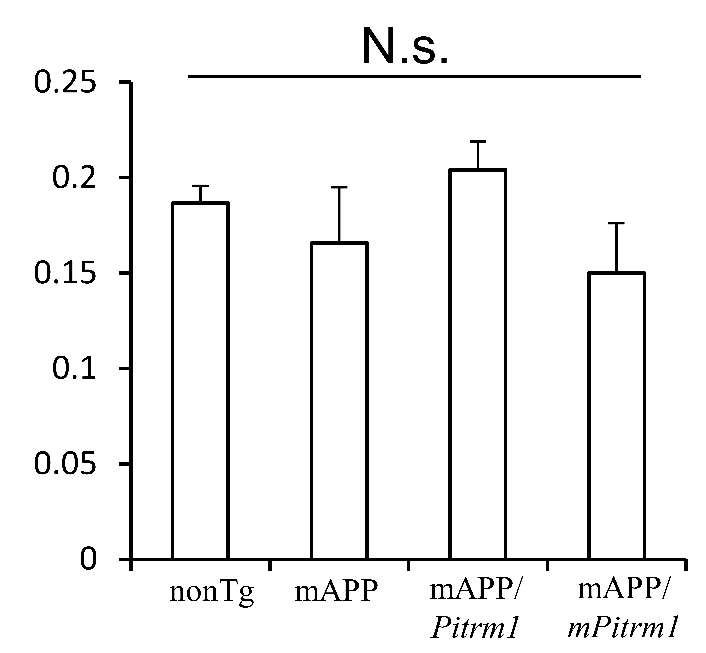


**Supplementary Figure 2.** Swimming speed of the indicated Tg mice in the Morris Water Maze test. The transgenic mice among these groups had similar swimming speed by the visual swimming speed test. N = 5-10 mice (3-6 male and 1-7 female per group) at age of 19-21 months.
